# Unveiling the spectrum of electrohydrodynamic turbulence in dust storms

**DOI:** 10.1038/s41467-023-36041-x

**Published:** 2023-01-25

**Authors:** Huan Zhang, You-He Zhou

**Affiliations:** 1 Key Laboratory of Mechanics on Disaster and Environment in Western China Attached to the Ministry of Education of China, Lanzhou University, Lanzhou, Gansu PR China; 2 Center for Particle-laden Turbulence, Lanzhou University, Lanzhou, Gansu PR China; 3 Department of Mechanics and Engineering Science, College of Civil Engineering and Mechanics, Lanzhou University, Lanzhou, Gansu PR China

**Keywords:** Atmospheric dynamics, Fluid dynamics

## Abstract

Although the electrical effects in dust storms have been observed for over 100 years, little is known about their fluctuating properties, especially for the dust concentration and electric fields. Here, using a combined observational and theoretical approach, we find that wind velocity, PM10 dust concentration, and electric fields in dust storms exhibit a universal spectrum when particle mass loading is low. In particular, all measured fields at and above 5 m display a power-law spectrum with an exponent close to − 5/3 in the intermediate-wavenumber range, consistent with the phenomenological theory proposed here. Below 5 m, however, the spectra of the wind velocity and ambient temperature are enhanced, due to the modulation of turbulence by dust particles at relatively large mass loading. Our findings reveal the electrohydrodynamic features of dust storms and thus may advance our understanding of the nonlinear processes in dust storms.

## Abstract (web summary)

Electrohydrodynamic features of dust storm turbulence have puzzled scientists for over a hundred years. Here, the authors reveal the characteristics of the multifield spectra in dust storms using a combined observational and theoretical approach.

## Introduction

Dust storms are typical atmospheric turbulence that is laden with massive dust particles. Due to particle electrification^1–5^, electric fields exceeding 100 kV m^−1^ are frequently produced during dust storms^6–13^, constituting a new distinct electrohydrodynamic (EHD) turbulence regime compared to ordinary turbulence^14,15^. As such, dust storms provide an opportunity to expand the knowledge on EHD turbulence at Reynolds number of up to ${{{{{{{\mathcal{O}}}}}}}}(1{0}^{6})$^16^, beyond that attainable through any laboratory experiment. Since the pioneering work of Rudge in 1913^6^, considerable effort has been made towards the exploration of the electrical properties of dust storms^6–13,17,18^, especially the mean electric fields are known to dramatically change the dynamics and transport of dust particles and wind fields^19,20^. Over the past century, however, the EHD statistical characteristics of the turbulent fluctuating fields have not yet been well understood, largely due to the difficulty in observing randomly occurred dust events and the high complexity of dust storms^21,22^.

For ordinary turbulent flows, velocity fields exhibit a universal Kolmogorov spectrum in the inertial range. In classical turbulence theory, energy is externally injected at the larger “outer” scale, and owing to nonlinear eddy interactions, such energy then successively decays into the smallest “inner” scale at which it is dissipated through molecular viscosity^23^. At sufficiently large Reynolds numbers, energy injection and dissipation scales are separated, and the scale invariance within the intermediate scales (i.e., inertial range) leads to a universal Kolmogorov *k*^−5/3^ power-law spectrum^24,25^. For passive scalars in turbulent flows, in addition to the Reynolds number, there exists a dimensionless number—Péclet number—which weighs the relative importance of velocity advection and scalar diffusivity. At high Reynolds and Péclet numbers, scalar fields exhibit a similar Richardson cascade process toward small scales, so that a phenomenological argument also results in a *k*^−5/3^ power-law scalar spectrum within the inertial-convective range^26–30^. These phenomenological descriptions are the celebrated Kolmogorov–Obukhov–Corrsin (KOC) arguments^25–27^.

However, the existence of such universal spectra in dust storm EHD turbulence remains unclear. Furthermore, particle-turbulence and particle-electrostatics couplings are substantial when particle mass loading is high^17–20,31^. These interphase couplings might alter the scaling properties of the spectrum^30,32^, but the details are unknown.

In this work, we carried out two months of field measurements within the atmospheric surface layer (ASL) to acquire high-quality data of wind velocity, ambient temperature, PM10 dust concentration (diameters ≤10 *μ*m), and electric fields in dust storms. To reveal the spectral characteristics, we evaluate the power spectral densities (PSDs) of the measured fields, which show how the fluctuating energies or variances are distributed across various scales^24–30^. To explain the measured PSDs, we further propose a phenomenological theory that assumes scale invariance and local-in-wavenumber-space interactions of PM10 dust concentrations and space-charge densities within the intermediate-wavenumber range.

## Results

### Overview of the datasets

The measurements were performed at an ASL turbulence observatory (39^∘^12′27″ N, 103^∘^40′03″ E) called the Qingtu lake observation array (QLOA), which is located between the Badain Jaran desert and the Tengger desert in China, with a dry, flat, erodible, and sandy surface of over 20 km^2^. The QLOA comprises 33 observation towers, allowing us to perform multipoint measurements. The wind velocities and ambient temperatures were measured using sonic anemometers (CSAT3B, Campbell Scientific) at ten heights ranging from 0.5 m to 12 m. The PM10 dust concentrations were measured using DustTrak II Aerosol Monitors (Model 8530EP, TSI Incorporated) at five heights ranging from 0.9 m to 12 m. The electric fields were measured using vibrating-reed electric field mills developed by Lanzhou University (see ref. ^17^ for the details) with ten components at eight locations with heights from 5 m to 8.5 m. The layout of the measurements is detailed in Supplementary Fig. 1. These instruments recorded data at a frequency of 50 Hz for wind velocities and ambient temperatures but 1 Hz for PM10 dust concentrations and electric fields. In addition, two dust collectors were mounted on 0.9 and 5 m heights to collect airborne dust particles during dust storms. Then, the number distributions of the collected dust samples were determined by a laser particle size analyzer (see Supplementary Fig. 2).

Although data were collected continuously from April to June, 2017, only a fraction of the data were used because the selected data had to satisfy two data-selection criteria: (1) long enough and statistically stationary; (2) near-neutral. Additionally, the synoptic scales that overlap with large-scale turbulent motions should be excluded (see Methods section). As a result, the resulting datasets were considered analogous to a flat-plate turbulent boundary layer^33,34^. In this study, three one-hour datasets derived from a particle-free windy condition, a mild dust storm (visibility greater than 0.3 km), and a severe dust storm (visibility less than 0.3 km) were regarded to be of high enough quality to be used in the PSD analysis.

The main parameters of the three selected datasets are summarized in Table 1. It is shown that the friction velocity, PM10 dust concentration, and electric field for the severe dust storm dataset are as high as 0.64 m s^−1^, 1.31 mg m^−3^, and 93.72 kV m^−1^, respectively, suggesting that the observed dust storm is very intense and electrically active. Meanwhile, four key particle parameters are presented in Table 2. Here, particle-to-air mass loading ratio Φ_*m*_^35,36^, Stokes number *St*^37^, electrostatic Stokes number *St*_*el*_^38^, and the ratio of the vertical terminal settling velocity of dust particles to the typical Lagrangian vertical air velocity *w*_*t*_/(*κu*_*τ*_)^39^, are used to quantify the importance of particle-turbulence coupling, particle inertia, electrostatic force, and gravitational settling, respectively.

**Table 1:** Summary of the main parameters of the selected three datasets

Parameter	Particle-free	Mild storm	Severe storm
Period	511.0023-0123	420.0930-1030	417.0508-0608
*ζ*	−2.56	−0.03	−0.03
RNP	0.08	0.14	0.13
*u*_*τ*_ (m s^−1^)	0.25	0.54	0.64
*U*_*c*_ (m s^−1^)	5.60	12.65	15.72
〈Θ〉 (K)	293.55	280.60	292.99
〈*C*〉 (mg m^−3^)	≲ 0.01	0.22	1.31
$\langle \left|{{{{{{{\bf{E}}}}}}}}\right|\rangle$ (kV m^−1^)	0.08	46.20	93.72
*χ* (km)	7.42	0.72	0.26

Here, the period, for example, “511.0023-0123” denotes time interval from 00:23:01 to 01:23:00 (UTC) on 11 May, 2017; *ζ* and RNP are the dimensionless Monin-Obukhov stability and relative stationary parameters, respectively (see Methods section); *u*_*τ*_ is the friction velocity, *U*_*c*_ is the mean convection velocity, 〈Θ〉 is the mean ambient temperature, 〈*C*〉 is the mean PM10 dust concentration, $\langle \left|{{{{{{{\bf{E}}}}}}}}\right|\rangle$ is the mean magnitude of the electric field, and *χ* denotes the visibility measured using a Belfort Model 6000 sensor at approximately 1 m above the surface. All parameters are calculated at 5 m above the surface, except for the visibility *χ*.

**Table 2:** Key particle parameters of the mild and severe dust storm datasets

Parameter	Mild storm	Severe storm
Φ_*m*_ at 0.9 m	0.04 ± 0.02	0.11 ± 0.05
Φ_*m*_ at 5 m	(1.07 ± 1.02) × 10^−4^	(6.38 ± 4.39) × 10^−4^
*St* at 0.9 m	0.99 ± 0.28	1.28 ± 0.36
*St* at 5 m	0.16 ± 0.12	0.21 ± 0.16
*St*_*el*_ at 0.9 m	0.010 ± 0.006	0.016 ± 0.011
*St*_*el*_ at 5 m	(5.24 ± 5.12) × 10^−5^	(1.28 ± 1.06) × 10^−4^
*w*_*t*_/(*κu*_*τ*_) at 0.9 m	0.61 ± 0.17	0.52 ± 0.15
*w*_*t*_/(*κu*_*τ*_) at 5 m	0.10 ± 0.07	0.08 ± 0.06

Here, Φ_m_ is the particle-to-air mass loading ratio, *St* is the particle Stokes number, *St*_*el*_ is the electrostatic Stokes number, and *w*_*t*_/(*κu*_*τ*_) is the ratio of the vertical terminal settling velocity of dust particles *w*_*t*_ to the typical Lagrangian vertical air velocity *κu*_*τ*_ (with *κ* and *u*_*τ*_ being the Von Kármán constant and friction velocity, respectively). The definition and estimation of these parameters are provided in the Methods section. Data are mean ± standard deviation.

For particles embedded in turbulent flows, the particle-to-fluid relative velocity is generally present owing to the finite response time of dust particles to velocity changes (i.e., inertial effects), electrostatic effects, and gravitational settling^40^. However, as demonstrated in previous works^35–39,41^, these effects for the dust particles at and above 5 m are believed to be negligible herein, because (1) the controlling parameters *St*, *St*_*el*_, and *w*_*t*_/(*κu*_*τ*_) are much less than unity at 5 m (see Table 2); (2) the mean diameters and concentrations of the dust particles decrease with height according to a power or logarithmic law^39,42^, leading to reductions of the controlling parameters with height (see Methods section). Even though the controlling parameters *St* and *w*_*t*_/(*κu*_*τ*_) at 0.9 m are on the order of ~0.1–1, they are also found to be very small for the PM10 particles^39^, suggesting that the inertia and gravitational settling of the PM10 particles are negligible and thus experience a long-term suspension. Accordingly, the particle-to-fluid relative velocity for the PM10 and all-sized dust particles at and above 5 m is nearly zero.

### Universality of PSDs

**Fig. 1:**
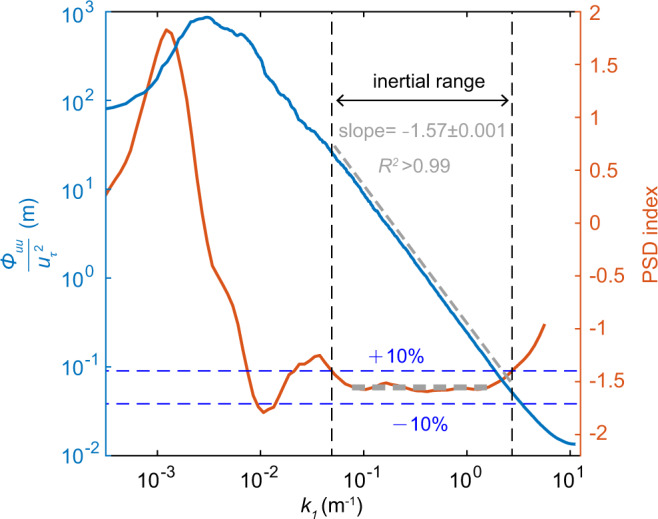
Example of determining the inertial range and the corresponding PSD scaling exponent. The data is the streamwise wind velocity at 12 m height for the mild dust storm dataset. The PSD of the streamwise velocity (left axis and blue line) is smoothed using a 25% bandwidth moving filter^60^ and divided by the squared friction velocity. The streamwise wavenumber *k*_1_ is obtained from the frequency *f* using Taylor’s frozen field hypothesis, i.e., *k*_1_ = 2*πf*/*U*_*c*_, where *U*_*c*_ represents the mean convection velocity. The PSD index (right axis and orange line) is determined by a best-fit method described in the main text. The horizontal dashed grey line marks the plateau of the PSD index, while the horizontal dashed blue lines mark the region that deviates from the plateau of the PSD index within ± 10%. The vertical dashed dark lines mark the corresponding inertial wavenumber range. The oblique dashed grey line denotes the best-fit line in the identified inertial wavenumber range, which is slightly shifted for clarity. The coefficient of determination *R*^2^ is larger than 0.99 and the fitted slope is − 1.57 ± 0.001 (95% confidence interval).

We use the fast Fourier transform (FFT)-based Welch’s method to compute PSDs^43^ (see Methods section). Figure 1 shows an example of the resulting PSD in log-log space. It can be seen that the PSD of the streamwise wind velocity appears to follow a power law, i.e., ${\phi }_{uu} \sim {k}_{1}^{\alpha }$, in the intermediate-wavenumber range. As done in ref. ^44^, the PSD index is determined by taking a sliding window of half a decade of wavenumber *k*_1_ over the PSD and calculating the best-fit linear gradient in the log-log space within this window. The inertial wavenumber range, *k*_1_ ∈ [0.049, 2.732], is thus identified as an interval that deviates from the plateau of the PSD index within ± 10%. The scaling exponent *α* = − 1.57 ± 0.001 (95% confidence interval) is finally determined by fitting the PSD linearly within the identified inertial range *k*_1_ ∈ [0.049, 2.732]. Using this approach, the resulting coefficients of determination *R*^2^ are larger than 0.99 for all PSD linear regressions, and the 95% confidence intervals for the fitted slopes are ~ 0.001 ( ~ 0.01) for 50 Hz (1 Hz) data, suggesting excellent power-law PSDs in the inertial wavenumber ranges.

**Fig. 2:**
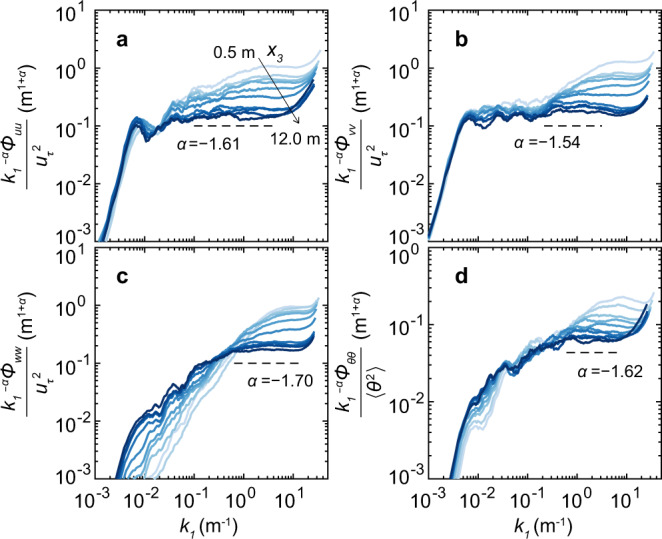
Compensated one-dimensional PSDs of the fluctuating fields for the observed particle-free dataset. **a**–**d** The compensated velocity PSDs (streamwise component ${k}_{1}^{-\alpha }{\phi }_{uu}$, spanwise component ${k}_{1}^{-\alpha }{\phi }_{vv}$, and vertical component ${k}_{1}^{-\alpha }{\phi }_{ww}$) and ambient temperature PSDs (${k}_{1}^{-\alpha }{\phi }_{\theta \theta }$) are divided by the squared friction velocity (i.e., ${u}_{\tau }^{2}$) and their variances (i.e., 〈*θ*^2^〉), respectively. The PSDs are smoothed using a 25% bandwidth moving filter^60^ and shown at ten heights from 0.5 m (light blue) to 12 m (dark blue). The scaling exponents of the PSDs, *α*, are determined by fitting the PSDs at 12 m height with a power function of the streamwise wavenumber *k*_1_, i.e., $\sim {k}_{1}^{\alpha }$ within the ranges of *k*_1_ denoted by the horizontal dashed lines.

For the particle-free dataset, the PSDs of the wind velocity and ambient temperature follow the expected Kolmogorov ${k}_{1}^{-5/3}$ scaling (with exponents within ~ 8% variations) in the inertial wavenumber range at all measured heights (see Fig. 2), but the inertial range becomes progressively narrower with decreasing height due to surface limitations^25–27,45,46^. The particle-free dataset is somewhat unstable, i.e., *ζ* = − 2.56 (see Table 1 and Methods section), but the buoyancy has a pronounced effect on the PSDs only in the low-wavenumber region adjacent to the near-neutral range, as confirmed by measurements^45^ and Claussen’s model^47,48^.

**Fig. 3:**
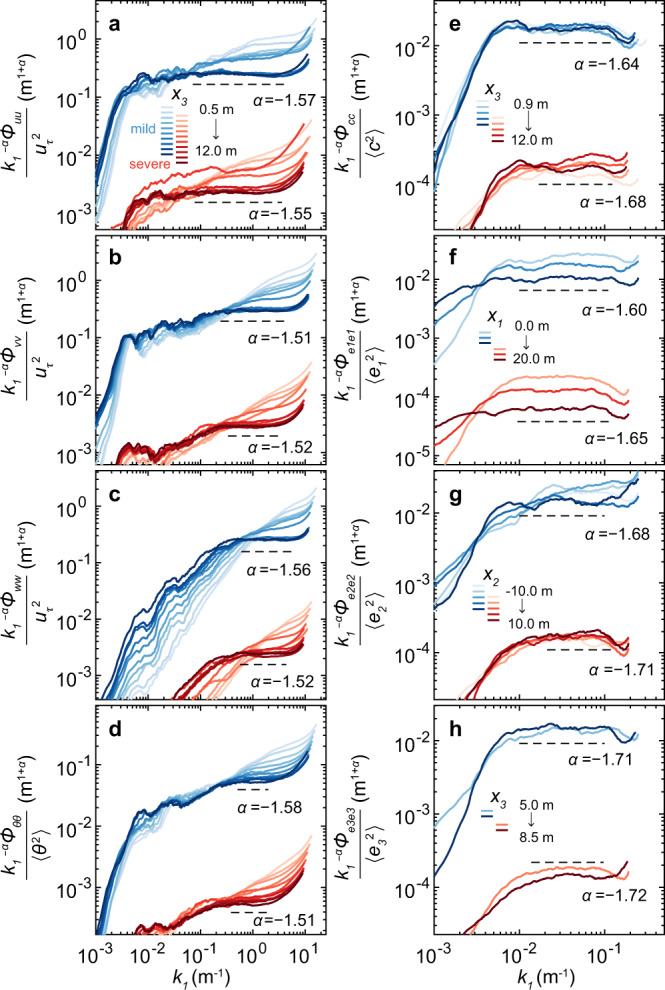
Compensated one-dimensional PSDs of the fluctuating fields for the observed mild (blue lines) and severe (red lines) dust storm datasets. **a**–**h** The compensated velocity PSDs (streamwise component ${k}_{1}^{-\alpha }{\phi }_{uu}$, spanwise component ${k}_{1}^{-\alpha }{\phi }_{vv}$, and vertical component ${k}_{1}^{-\alpha }{\phi }_{ww}$) are divided by the squared friction velocity (${u}_{\tau }^{2}$), whereas the compensated PSDs of the ambient temperatures (${k}_{1}^{-\alpha }{\phi }_{\theta \theta }$), PM10 dust concentrations (${k}_{1}^{-\alpha }{\phi }_{cc}$), and electric fields (streamwise component ${k}_{1}^{-\alpha }{\phi }_{{e}_{1}{e}_{1}}$, spanwise component ${k}_{1}^{-\alpha }{\phi }_{{e}_{2}{e}_{2}}$, and vertical component ${k}_{1}^{-\alpha }{\phi }_{{e}_{3}{e}_{3}}$) are divided by their variances, respectively. These results are smoothed using a 25% bandwidth moving filter^60^ and shown at ten heights from *x*_3_ = 0.5 m to *x*_3_ = 12 m and three streamwise locations from *x*_1_ = 0 m to *x*_1_ = 20 m, as well as five spanwise locations from *x*_2_ = − 10 m to *x*_2_ = 10 m. Here, *x*_1_, *x*_2_, and *x*_3_ are the streamwise, spanwise, and vertical space coordinates, respectively. The scaling exponents of the PSDs, *α*, are determined by fitting the PSDs at the maximum *x*_1_-, *x*_2_-, and *x*_3_-coordinate with a power function of the streamwise wavenumber *k*_1_, i.e., $\sim {k}_{1}^{\alpha }$ within the ranges of *k*_1_ denoted by the horizontal dashed lines. For clarity, the compensated PSDs for the severe dust storm dataset are vertically shifted by 10^−2^ in logarithmic scale.

Nonetheless, for both mild and severe dust storm datasets, the PSDs of the wind velocity and ambient temperature only follow the ${k}_{1}^{-5/3}$ scaling well at 5–12 m in the inertial wavenumber range ~ 5 × 10^−2^-10^0^ m^−1^, except for *v*-, *w*-, and *θ*-PSDs having a relatively narrow range. This wavenumber range corresponds to a plateau in the compensated PSDs (denoted by the horizontal dashed lines in Fig. 3), where the scaling exponents *α* are within ~ 10% variations of − 5/3 (see Fig. 3a–d).

By contrast, below 5 m, these PSDs are enhanced and deviate from the ${k}_{1}^{-5/3}$ scaling more and more significantly with decreasing heights. As shown in Table 2, the particle-to-air mass loading ratio Φ_*m*_ at 0.9 m (5 m) height are estimated to be on the order of ~ 0.1 ( ≲ 10^−4^), suggesting a strong (weak) two-way particle-turbulence coupling^35,36,49^. Consequently, we can reasonably infer that such PSD enhancements in the inertial range may arise from the modulation of wind velocity due to massive loads of dust particles below 5 m^36^. Since the total concentration of the dust particles decreases rapidly with increasing height^39^, wind modulation by dust particles becomes negligible at larger heights.

For the PM10 dust concentration and electric fields, the PSDs follow ${k}_{1}^{-5/3}$ scaling fairly well (within ~ 4% variations) in the wavenumber range ~ 10^−2^-10^−1^ m^−1^ (corresponding to the plateaus in Fig. 3e, f) at different locations. Notably, the PSD enhancements were not observed because the sampling frequency of the PM10 dust concentrations and electric fields was only 1 Hz.

It is worthwhile to note that at the large-wavenumber end of the spectra, all PSDs seem to exhibit other distinct scalings. However, the evidence for such distinct scalings is not completely convincing because the corresponding wavenumber interval is too short and is very close to the noise level of the measurements.

### Phenomenological theory

To interpret the Kolmogorov scaling of the PM10 dust concentrations and electric fields in the intermediate-wavenumber range, we formulated a phenomenological theory analogous to the KOC arguments^25–30^. A detailed description of our theory is offered in Supplementary Information, but we describe it here briefly. Let *ψ* be the transported fluctuating scalar field, such as PM10 dust concentration and space-charge density. According to the variance budget equation of the scalar field without considering particle-to-fluid relative velocity, the mean dissipation rate of the scalar variance, *ε*, can be defined as^50^:

1 $$\varepsilon=2\varGamma \left\langle \frac{\partial \psi }{\partial {x}_{j}}\frac{\partial \psi }{\partial {x}_{j}}\right\rangle ,$$

where *Γ* represents the molecular diffusivity of the scalar field, the brackets 〈 ⋅ 〉 denotes the Reynolds average, and *x*_*j*_ ( *j* = 1, 2, 3) is the *j*th component of the space coordinates. Here and henceforth, the Einstein summation convention is applied for space coordinates.

Assuming that there also exist similar Richardson cascade processes for the variances of the PM10 dust concentrations and space-charge densities, this brings out two assumptions in the intermediate-wavenumber range^51^. The first is scale invariance, i.e., the variance flux is independent of the wavenumber and equal to the mean variance dissipation rate *ε*. The second is the local-in-wavenumber-space interaction, i.e., the variance flux occurs only between eddies of similar scales. Meanwhile, because the PM10 dust concentration fluctuations are closely related to velocity fluctuations, the PSD of the PM10 dust concentration *ϕ*_*c*_(*k*) is thus determined by the PM10 dust concentration dissipation rate *ε*_*c*_ = *Γ*_*c*_〈(∂*c*/∂*x*_*j*_)(∂*c*/∂*x*_*j*_)〉, the wavenumber *k*, and the mean turbulent energy dissipation rate *ε*_*t*_ = *ν*〈(∂*u*_*i*_/∂*x*_*j*_)(∂*u*_*i*_/∂*x*_*j*_)〉, where *Γ*_*c*_ represents the molecular diffusivity of the PM10 dust concentration, and *ν* represents the kinematic viscosity of the air. Similarly, the PSD of the space-charge density *ϕ*_*ρ*_(*κ*) depends on the space-charge density dissipation rate *ε*_*ρ*_ = *Γ*_*ρ*_〈(∂*ρ*/∂*x*_*j*_)(∂*ρ*/∂*x*_*j*_)〉, the wavenumber *k*, and *ε*_*t*_, where *Γ*_*ρ*_ represents the molecular diffusivity of space-charge density. Therefore, the dimensional analysis gives (see Supplementary Information for the details):

2a $${\phi }_{c}(\kappa ) \sim {\varepsilon }_{c}{\varepsilon }_{t}^{-\frac{1}{3}}{k}^{-\frac{5}{3}} ,$$

2b $${\phi }_{\rho }(\kappa ) \sim {\varepsilon }_{\rho }{\varepsilon }_{t}^{-\frac{1}{3}}{k}^{\frac{1}{3}}.$$

To obtain the PSD of the electric field, we must determine the scaling relation between the electric field and the space-charge density. According to the Gauss’s law^52^, we have

3 $$\frac{{\partial }^{2}\varphi ({{{{{{{\bf{x}}}}}}}})}{\partial {x}_{j}\partial {x}_{j}}=-\frac{\rho ({{{{{{{\bf{x}}}}}}}})}{{\varepsilon }_{0}};\quad {e}_{j}({{{{{{{\bf{x}}}}}}}})=-\frac{\partial \varphi ({{{{{{{\bf{x}}}}}}}})}{\partial {x}_{j}} ,$$

where *φ*(**x**) and *e*_*j*_(**x**) represent the fluctuating electric potential and electric field at the position vector **x**, respectively, and *ε*_0_ represents the permittivity of the vacuum. Based on Eq. (3), Gauss’s law in the wavenumber space can be written as^52^:

4 $$\hat{{e}_{j}}({{{{{{{\bf{k}}}}}}}})=-i{k}_{j}\frac{\hat{\rho }({{{{{{{\bf{k}}}}}}}})}{{k}^{2}{\varepsilon }_{0}} ,$$

where $i=\sqrt{-1}$ represents the imaginary unit, $\hat{{e}_{j}}({{{{{{{\bf{k}}}}}}}})$ and $\hat{\rho }({{{{{{{\bf{k}}}}}}}})$ represent the Fourier modes of *e*_*j*_(**x**) and *ρ*(**x**) at wavenumber vector **k**, respectively, and $k=\left|{{{{{{{\bf{k}}}}}}}}\right|$ represents the magnitude of the wavenumber vector. From Eq. (4), the spectrum tensor of the electric field Φ_*e*,*lm*_(**k**) is related to that of the space-charge density Φ_*ρ*_(**k**) by:

5 $${\Phi }_{e ,lm}({{{{{{{\bf{k}}}}}}}})=\frac{{k}_{l}{k}_{m}}{{k}^{4}{\varepsilon }_{0}^{2}}{\Phi }_{\rho }({{{{{{{\bf{k}}}}}}}}) ,$$

where the subscripts *l* and *m* ∈ {1, 2, 3} also denote the space coordinates.

The PSD is obtained from the spectrum tensor by integrating over all the wavenumbers **k** of magnitude *k*. Therefore, we have:

6 $${\phi }_{e}(k)=\frac{1}{{k}^{2}{\varepsilon }_{0}^{2}}{\phi }_{\rho }(k).$$

By combining Eq. (2b) and Eq. (6), we obtain the desired Kolmogorov scaling for the PSD of the electric field in the intermediate-wavenumber range:

7 $${\phi }_{e}(k) \sim {k}^{-2+\frac{1}{3}} \sim {k}^{-\frac{5}{3}}.$$

As shown in Eq. (2a) and Eq. (7), we obtain a universal − 5/3 power-law spectrum for the PM10 dust concentrations and electric fields within the intermediate-wavenumber range. By using the relation between the one- and three-dimensional spectra, we find that one-dimensional spectra also scale with ${k}_{1}^{-5/3}$ (see Supplementary Information), which is in line with our measured results.

## Discussion

In summary, we discover and describe the spectral features of the fluctuating fields in dust storms, both experimentally and theoretically. Based on the experimental results, we demonstrate that under stationary and near-neutral conditions, all PSDs at and above 5 m show a universal power-law with an exponent close to − 5/3 within the intermediate-wavenumber range. This conclusion is drawn from two dust storm datasets, whose reliability would be enhanced with additional datasets. The multifield spectral features under nonstationary and non-neutral conditions are unclear and warrant further investigations. In addition, below 5 m, the PSDs of the wind velocity and ambient temperature are enhanced in the intermediate-wavenumber range due to turbulence modulation by massive loads of dust particles, suggesting that the wind velocity and ambient temperature should no longer behave as classic wall turbulence at these scales.

To elucidate such a universal PSD, we introduce a phenomenological theory based on the Kolmogorov-style analysis of the local-in-wavenumber-space cascade of the variances of PM10 dust concentration and space-charge density. The particle-to-fluid relative velocity were not considered here because particle inertia, electrostatic force, and gravitational settling are negligible. By holding the assumptions of scale invariance and local-in-space interactions, the standard dimensional analysis predicts *ϕ*_*c*_ ~ *k*^−5/3^, but *ϕ*_*ρ*_ ~ *k*^1/3^. Additionally, because *ϕ*_*ρ*_ ~ *k*^−2^*ϕ*_*e*_, we have *ϕ*_*e*_ ~ *k*^−5/3^. This suggests a fluid-like nonlinear cascade picture of the PM10 dust concentration and electric field, resulting from different physical processes.

Note that the inertial ranges of the PM10 dust concentrations and electric fields are extended to the lower wavenumbers compared to the velocity fields. Future work is needed to shed light on the spectral characteristics at high wavenumbers and to explore how the dust particles modulate atmospheric turbulence at large mass loading.

## Methods

### Data qualification

To obtain usable data, the collected raw data should be qualified through the following data-qualification procedures^34^. First of all, the selected dataset had to be statistically stationary, such that the time-averaged values were equivalent to the corresponding ensemble-averaged values^53^. Additionally, the dataset had to be long enough to obtain converged statistics on the low wavenumber events in the atmospheric surface layer (ASL). To eliminate the effects of buoyancy, the dataset was also required to have near-neutral conditions with negligible heat fluxes to or from the wall. Importantly, because the large-scale coherent motions in the ASL overlap with weather-related phenomena, the synoptic scales had to be filtered out of the raw data^34^.

First, the stationarity of the time series {*Y*(*n*) : *n* = 0, …, *N* − 1} is characterised using the relative non-stationarity parameter (RNP), as follows^53,54^:

8 $${{{{{{{\rm{RNP}}}}}}}}=\left|1-\frac{\mathop{\sum }\nolimits_{j=0}^{M-1}\langle {\,y}_{j}^{2}\rangle }{M\langle {\,y}^{2}\rangle }\right|,$$

where the time series {*Y*(*n*)} is equally divided into *M* contiguous segments {*Y*_*j*_(*n*) = *Y*( *jN*/*M*, …, ( *j* + 1)*N*/*M* − 1) :  *j* = 0, …, *M* − 1} of approximately 5 min, $\langle {\,y}_{j}^{2}\rangle$ represents the variance of the *j*th segment, and 〈*y*^2^〉 represents the variance of the entire time series. Clearly, the RNP represents the variations of the time series over time. If the RNP is less than 0.3, the time series is considered stationary^54^.

Second, the data must fulfil the near-neutral conditions, such that the influence of thermal stratification is negligible. The stability of the data can be examined using the dimensionless Monin-Obukhov stability parameter:

9 $$\zeta=-\frac{{x}_{3}\kappa g\langle {(w\Theta )}_{0}\rangle }{\langle \Theta \rangle {u}_{\tau }^{3}} ,$$

where *x*_3_ represents the height above the surface, *L* represents the Obukhov length, *κ* = 0.41 represents the von Kármán constant, *g* = 9.81 ms^−2^ represents the gravitational acceleration, 〈(*wθ*)_0_〉 represents the surface heat flux, 〈Θ〉 represents the mean temperature, and ${u}_{\tau }={({\langle uw\rangle }^{2}+{\langle vw\rangle }^{2})}^{1/4}$ represents the friction velocity calculated at *x*_3_ = 2.5 m. Typically, when $\left|\zeta \right|\lesssim 0.1$, the ASL at height *x*_3_ is considered near-neutral^46,55^.

Third, the data must be de-trended because the large-scale coherent structures in ASLs reach 10–20 times the surface-layer thickness, which overlaps with the synoptic scales. Since the events covering the entire measurement domain are considered weather related^34^, synoptic scales can be removed in the following ways: (1) for fluctuating fields { *y* ^*j*^(*n*) : *n* = 0, …, *N* − 1; *j* = 0, …, *Q* − 1} measured at *Q* different locations, the location-averaged time series is defined as

10 $${\langle \,y(n)\rangle }_{j}=\frac{\mathop{\sum }\nolimits_{j=0}^{Q-1}{y}^{\,j}(n)}{Q} ,$$

where *n* = 0, …, *N* − 1. (2) the location-averaged time series is then low-pass filtered with a cut-off wavelength of 1.5 km to extract the synoptic scale { *y*_*s*_(*n*) : *n* = 0, …, *N* − 1}; and finally (3) the desired fluctuating data are de-trended by subtracting this synoptic scale from the raw fluctuating data, i.e.,

11 $$y(n)=y(n)-{y}_{s}(n) ,$$

where *n* = 0, …, *N* − 1. Note that, at different locations, the same components of the electric field may be directed oppositely^13^. In such cases, the location-averaged time series should be modified as

12 $${\langle \,y(n)\rangle }_{j}=\frac{\mathop{\sum }\nolimits_{j=0}^{Q-1}{{{{{{{\rm{sgn}}}}}}}}(\langle {Y}^{j}(n)\rangle ){y}^{\,j}(n)}{Q} ,$$

where *n* = 0, …, *N* − 1, and ${{{{{{{\rm{sgn}}}}}}}}(\cdot )$ represents the sign function. Accordingly, to de-trend correctly, the desired fluctuating data are obtained using the following:

13 $$y(n)=y(n)-{{{{{{{\rm{sgn}}}}}}}}(\langle {Y}^{j}(n)\rangle ){y}_{s}(n).$$

where *n* = 0, …, *N* − 1. Apart from the data qualifications mentioned above, there is no further data processing.

As an example, the extracted fluctuating time series of the severe dust storm dataset is presented in Supplementary Fig. 3.

### Key particle parameters

To assess whether particle inertia, electrostatic forces, and gravitational settling are negligible in the observed dust storm datasets, we quantified three key particle parameters. First, the Stokes number *St*, which is defined as the ratio of the particle relaxation time scale *τ*_*p*_ to the Kolmogorov time scale *τ*_*η*_,

14 $$St=\frac{{\tau }_{p}}{{\tau }_{\eta }} ,$$

is a measure of the effects of inertia on particle dynamics.

In the cases considered herein, because particle Reynolds number is less than unity (i.e., the Stokes regime) and particles are much denser than the fluid (see refs. ^39,56^ for the details), particle relaxation time scale *τ*_*p*_ can be computed as^37^

15 $${\tau }_{p}=\frac{{\rho }_{p}{d}_{p}^{2}}{18\nu {\rho }_{a}} ,$$

where *ρ*_*p*_ and *ρ*_*a*_ are mass densities of the dust particles and air, respectively; *d*_*p*_ is particle diameter; *ν* is the kinematic viscosity of the air.

The Kolmogorov time scale *τ*_*η*_ can be estimated as follows in the log-law region^24^

16 $$\left\{\begin{array}{l}{\tau }_{\eta }=\frac{{\eta }^{2}}{\nu } ,\hfill \\ \frac{\eta }{{\delta }_{\nu }}={(\kappa {x}_{3}^{+})}^{1/4} ,\quad \end{array}\right.$$

where *η* is the Kolmogorov microscale, *κ* = 0.41 is the Von Kármán constant, *δ*_*ν*_ = *ν*/*u*_*τ*_ is the viscous lengthscale, *u*_*τ*_ is the friction velocity, and ${x}_{3}^{+}={x}_{3}/{\delta }_{\nu }$ is the dimensionless height measured in viscous lengthscale.

It is well recognized that particles are expected to be quasi-ballistic when *St* ≫ 1, while they are expected to strictly follow the fluid when *St* ≪ 1^37^.

Second, the effects of electrostatic force on the dust particles can be quantified by the electrostatic Stokes number *St*_*el*_, which is defined as^38^

17 $$S{t}_{el}=\frac{{\tau }_{p}}{{\tau }_{el}}.$$

Here, the characteristic time scale of electrostatic interactions *τ*_*el*_ is defined as

18 $${\tau }_{el}=\frac{1}{{Q}_{p}}\sqrt{\frac{3{m}_{p}}{2\lambda {n}_{p}}} ,$$

with *Q*_*p*_ and *m*_*p*_ being the electric charge and mass of the particle, respectively, *n*_*p*_ being the mean particle number density, *λ* = 1/(4*πϵ*_0_), and *ϵ*_0_ = 8.854 × 10^−12^ F m^−1^ being the vacuum permittivity.

As reported in Ref. ^38^, the electrostatic effects on particle dynamics are negligible when *St*_*el*_ ≪ 1, while they are dominant when *St*_*el*_ ≫ 1.

Third and finally, in the Stokes regime the vertical terminal velocity *w*_*t*_ due to gravitational settling is given as^39,56^

19 $${w}_{t}={\tau }_{p}g.$$

When the vertical terminal settling velocity of the particles is comparable to or less than the mean Lagrangian vertical velocity of the air parcel containing the particles (Lagrangian velocity is the velocity at which air parcels are dispersed upward by turbulence), the particles would remain suspended. In a neutral ASL, the typical Lagrangian vertical velocity is approximately *κu*_*τ*_^57^. Therefore, the effects of the gravitational settling is believed to be negligible when *w*_*t*_/(*κu*_*τ*_) ≪ 1^39^.

Besides the mass-loading ratio Φ_*m*_, the parameters *St*, *St*_*el*_, and *w*_*t*_/(*κu*_*τ*_) are estimated based on the synchronous measurements of the wind velocities, PM10 dust concentrations, and particle size distributions (see Supplementary Fig. 2). In this study, particle mass density is assumed to be 1000 kg m^−3^; the density and kinematic viscosity of the air are taken as 1.20 kg m^−3^ and 1.57 × 10^−5^ m^2  ^s^−1^, respectively; the electric charge on dust particle, *Q*_*p*_, is calculated by the product of the particle’s charge-to-mass ratio and mass. Here, the magnitude of the charge-to-mass ratio of the particle is taken as 60 *μ*C kg^−1^, which is consistent with the measurements^58,59^.

### Power spectrum estimation

We use the FFT-based Welch’s method to estimate the PSD and cross-PSD^43^. Here, the fluctuating time series { *y*(*n*) : *n* = 0, …, *N* − 1} is divided into *M* disjoint segments {*y*_*j*_(*n*) = *y*(*n* + *jD*), *n* = 0, …, *L* − 1; *j* = 0, …, *M* − 1} with *L* − *D* overlaps between two adjacent segments, after which each segment is windowed using a Hamming window $\{W(n)=0.54-0.46\cos (2\pi n/(L-1)):n=0 ,\ldots ,\,L-1\}$, and finally, the modified periodograms for these segments are averaged to obtain the PSD estimator. The modified periodogram of segment {*y*_*j*_(*n*)} at frequency *f* is calculated as:

20 $${\phi }_{y ,\,j}(\;f)=\frac{1}{PL{\delta }_{t}}{\left|\,{\widetilde{y}}_{j}(\,f ,L)\right|}^{2} ,$$

with

21a $${\widetilde{y}}_{j}(\;f ,L)={\delta }_{t}\mathop{\sum }\limits_{n=0}^{L-1}W(n){y}_{j}(n){e}^{-i2\pi fn{\delta }_{t}} ,$$

21b $$P=\frac{1}{L}\mathop{\sum }\limits_{n=0}^{L-1}|W(n){|}^{2} ,$$

where *δ*_*t*_ represents the sampling interval of the time series { *y*(*n*)}. The Welch’s estimator of PSD is then determined by averaging the modified periodograms:

22 $${\phi }_{y}(f)=\frac{1}{M}\mathop{\sum }\limits_{j=0}^{M-1}{\phi }_{y ,\,j}(\;f).$$

Here, the time series {*y*(*n*)} is divided into eight segments with 50% overlap, and the Welch’s estimators are efficiently calculated using the FFT.

## Supplementary information

Supplementary Information

## Data Availability

All spectral data presented in this study have been deposited in the figshare repository and are available at 10.6084/m9.figshare.20655255.v1.

## Code availability

The code that support the findings of this study is available upon request to the corresponding author.
