# Supplementary material for: Unveiling the spectrum of electrohydrodynamic turbulence in dust storms
Source: Nat Commun. 2023 Jan 25;14:408. doi: 10.1038/s41467-023-36041-x (PMC9876929; doi:10.1038/s41467-023-36041-x)
Supplement: Supplementary file 1 — Supplementary Information [file 41467_2023_36041_MOESM1_ESM.pdf]

Supplemental Information for "Unveiling the spectrum of electrohydrodynamic turbulence in dust storms"

Huan Zhang,[1,2,3] You-He Zhou[1,3,*]

[1] Key Laboratory of Mechanics on Disaster and Environment in Western China Attached to the Ministry of Education of China, Lanzhou University, Lanzhou, Gansu 730000, PR China

[2] Center for Particle-laden Turbulence, Lanzhou University, Lanzhou, Gansu 730000, PR China

[3] Department of Mechanics and Engineering Science, College of Civil Engineering and Mechanics, Lanzhou University, Lanzhou, Gansu 730000, PR China

[*] Corresponding author: zhouyh@lzu.edu.cn

I. DERIVATION OF THE UNIVERSAL SPECTRA

A. Variance budget equation

Let $\mathit{\Psi}$ be the transported scalar fields, such as PM10 dust concentration and space-charge density (charge per unit volume). In contrast to the transport equation of passive scalar, the scalar transport equation associated with dust particles without a source or sink is given as[1,2]

$$\frac{\partial \mathit{\Psi}}{\partial t}+\frac{\partial (U_j+U_j^r)\mathit{\Psi}}{\partial x_j}=\mathit{\Gamma}\frac{\partial^2 \mathit{\Psi}}{\partial x_j \partial x_j}, \tag{1}$$

where U_j ($j=1,2,3$) is the jth component of the wind velocity, U_j^r is the particle-to-fluid relative velocity, and $\mathit{\Gamma}$ is the molecular diffusivity of the scalar $\mathit{\Psi}$. Hereafter, the Einstein summation convention is applied for space coordinates, i.e., $j \in \{1,2,3\}$.

As discussed in the main text, the particle-to-fluid relative velocity is approximately $U_j^r \approx 0$. In this case, the variance budget equation of the scalar $\mathit{\Psi}$ can be written as[3]

$$\frac{\partial \langle \psi^2\rangle}{\partial t}+\langle U_j\rangle \frac{\partial \langle \psi^2\rangle}{\partial x_j}=-2\langle \psi u_j\rangle \frac{\partial \langle \mathit{\Psi}\rangle}{\partial x_j}-\frac{\partial \langle \psi^2 u_j\rangle}{\partial x_j} +\mathit{\Gamma}\frac{\partial^2\langle \psi^2\rangle}{\partial x_j \partial x_j}-2\mathit{\Gamma}\left\langle \frac{\partial \psi}{\partial x_j}\frac{\partial \psi}{\partial x_j}\right\rangle. \tag{2}$$

B. Power spectral density of the PM10 dust concentration and space-charge density

The last term in Eq. (2) is always negative, thereby resulting in a decrease in the variance $\langle \psi^2\rangle$ with time. Therefore, the mean dissipation rate of the variances of the PM10 dust concentration, ϵ_c, and space-charge density, ϵ_ρ, are respectively defined as[4,5]

$$\epsilon_c = 2\mathit{\Gamma}_c\left\langle \frac{\partial c}{\partial x_j}\frac{\partial c}{\partial x_j}\right\rangle, \tag{3a}$$

$$\epsilon_\rho = 2\mathit{\Gamma}_\rho\left\langle \frac{\partial \rho}{\partial x_j}\frac{\partial \rho}{\partial x_j}\right\rangle, \tag{3b}$$

where c and ρ are the fluctuating PM10 dust concentration and space-charge density, respectively. Assuming that ϕ_c is only determined by ϵ_c, turbulent energy dissipation rate $\varepsilon_t=\nu\langle(\partial u_i/\partial x_j)(\partial u_i/\partial x_j)\rangle$, and wavenumber k, while ϕ_ρ depends only on ϵ_ρ, ϵ_t, and k in the intermediate-wavenumber range, we have

$$\phi_c(k)=A\epsilon_c^{\alpha_1}\epsilon_t^{\beta_1}k^{\gamma_1}, \tag{4a}$$

$$\phi_\rho(k)=B\epsilon_\rho^{\alpha_2}\epsilon_t^{\beta_2}k^{\gamma_2}, \tag{4b}$$

where A and B are dimensionless constants. The dimension of the related quantities in Eq. (4a) and Eq. (4b) are

$$\begin{aligned}
[\phi_c] &= \frac{M^2}{L^5},\\
[\phi_\rho] &= \frac{I^2T^2}{L^5},\\
[\epsilon_t] &= \frac{L^2}{T^3},\\
[\epsilon_c] &= \frac{M^2}{L^6T},\\
[\epsilon_\rho] &= \frac{I^2T}{L^6},\\
[k] &= \frac{1}{L},
\end{aligned} \tag{5}$$

where the square brackets $[\cdot]$ denote "the dimension", $M \equiv$ "Mass", $L \equiv$ "length", $I \equiv$ "Current", and $T \equiv$ "Time". Hence, the dimensional analysis gives

$$\frac{M^2}{L^5}=\left(\frac{M^2}{L^6T}\right)^{\alpha_1}\left(\frac{L^2}{T^3}\right)^{\beta_1}\left(\frac{1}{L}\right)^{\gamma_1}, \tag{6a}$$

$$\frac{I^2T^2}{L^5}=\left(\frac{I^2T}{L^6}\right)^{\alpha_2}\left(\frac{L^2}{T^3}\right)^{\beta_2}\left(\frac{1}{L}\right)^{\gamma_2}. \tag{6b}$$

Solving Eq. (6a) and Eq. (6b), we obtain $\alpha_1=1$, $\beta_1=-1/3$, and $\gamma_1=-5/3$; $\alpha_2=1$, $\beta_2=-1/3$, and $\gamma_2=1/3$, thus Eq. (4a) and Eq. (4b) can be explicitly written as

$$\phi_c(k) = A\varepsilon_c\varepsilon_t^{-\frac{1}{3}}k^{-\frac{5}{3}}, \tag{7a}$$

$$\phi_\rho(k) = B\varepsilon_\rho\varepsilon_t^{-\frac{1}{3}}k^{\frac{1}{3}}. \tag{7b}$$

C. Power spectral density of the electric field

The space-charge density is related to the electric field by the celebrated Gauss's law

$$\frac{\partial^2\varphi(\mathbf{x})}{\partial x_j\partial x_j} = -\frac{\rho(\mathbf{x})}{\varepsilon_0}; \quad e_j(\mathbf{x}) = -\frac{\partial\varphi(\mathbf{x})}{\partial x_j}, \tag{8}$$

where $\varphi(\mathbf{x})$ and $e_j(\mathbf{x})$ are the fluctuating electric potential and the jth component of the electric field at the position vector $\mathbf{x}$, respectively, as well as ε_0 is the permittivity of the vacuum. The Gauss's law in wavenumber space can then be written as[6]

$$\hat{e_j}(\mathbf{k}) = -ik_j\frac{\hat{\rho}(\mathbf{k})}{k^2\varepsilon_0}, \tag{9}$$

where $i = \sqrt{-1}$ is the imaginary unit, $\hat{(\cdot)}$ denotes the Fourier model, and $k = |\mathbf{k}|$ is the magnitude of the wavenumber vector $\mathbf{k}$.

According to Eq. (9), the covariance between two Fourier modes $\hat{R}_{e,lm}(\mathbf{k})$ and $\hat{R}_\rho(\mathbf{k})$ are related via

$$\begin{aligned}\hat{R}_{e,lm}(\mathbf{k}) &\equiv \left\langle\overline{\hat{e_l}(\mathbf{k})}\hat{e}_m(\mathbf{k})\right\rangle, \\ &= \frac{k_lk_m}{k^4\varepsilon_0^2}\left\langle\overline{\hat{\rho}_l(\mathbf{k})}\hat{\rho}_m(\mathbf{k})\right\rangle, \\ &= \frac{k_lk_m}{k^4\varepsilon_0^2}\hat{R}_\rho(\mathbf{k}),\end{aligned} \tag{10}$$

where $\overline{(\cdot)}$ denotes the complex conjugate, as well as $l \in \{1,2,3\}$ and $m \in \{1,2,3\}$ corresponding to the space coordinates. The spectrum tensors are defined by[7]

$$\begin{aligned}\Phi_\rho(\mathbf{k}) &= \sum_{\mathbf{k}'}\delta(\mathbf{k}-\mathbf{k}')\hat{R}_\rho(\mathbf{k}'), \\ \Phi_{e,lm}(\mathbf{k}) &= \sum_{\mathbf{k}'}\delta(\mathbf{k}-\mathbf{k}')\hat{R}_{e,lm}(\mathbf{k}'),\end{aligned} \tag{11}$$

where $\delta(\cdot)$ is the three-dimensional delta function. Combining Eq. (10) and Eq. (11), the spectrum tensor of the electric field $\Phi_{e,lm}(\mathbf{k})$ is related to the spectrum tensor of the space-charge density $\Phi_\rho(\mathbf{k})$ by

$$\Phi_{e,lm}(\mathbf{k}) = \frac{k_lk_m}{k^4\varepsilon_0^2}\Phi_\rho(\mathbf{k}). \tag{12}$$

The spectrum $\phi(k)$ is obtained from the spectrum tensor by integrating over all wavenumbers $\mathbf{k}$ of magnitude k, which gives[7]

$$\phi(k) = \oint\frac{1}{2}\Phi_{ll}(\mathbf{k})dS(k), \tag{13}$$

where $dS(k)$ is an area element on the sphere $|\mathbf{k}| = k$. Substituting Eq. (12) into Eq. (13), we get the relation

$$\begin{aligned}\phi_e(k) &= \frac{k_lk_l}{k^4\varepsilon_0^2}\oint\frac{1}{2}\Phi_p(\mathbf{k})dS(k), \\ &= \frac{1}{k^2\varepsilon_0^2}\phi_\rho(k).\end{aligned} \tag{14}$$

Combining Eq. (7b) and Eq. (14), we obtained the desired scaling relation for the spectrum of the electric field in the intermediate-wavenumber range

$$\phi_e(k) \sim k^{-2+\frac{1}{3}} \sim k^{-\frac{5}{3}}. \tag{15}$$

D. One-dimensional power spectral density

Since the statistical properties of the PM10 dust concentration and electric field are assumed to be spatially homogeneous and isotropic in the intermediate-wavenumber range, the one-dimensional spectrum, ϕ_{11}, ϕ_{22}, and ϕ_{33}, can be expressed in terms of the spectrum $\phi(k)$ as[7]

$$\phi_{11}(k_1) = \frac{1}{2}\int_{k_1}^{\infty}\frac{\phi(k)}{k}\left(1-\frac{k_1^2}{k^2}\right)dk, \tag{16a}$$

$$\phi_{22}(k_1) = \phi_{33}(k_1) = \frac{1}{4}\int_{k_1}^{\infty}\frac{\phi(k)}{k}\left(1+\frac{k_1^2}{k^2}\right)dk. \tag{16b}$$

Substituting $\phi(k) \sim k^{-5/3}$ into Eq. (16a) and Eq. (16b), we find that the one-dimensional spectra also follow power-law with the same index:

$$\phi_{11}(k) \sim k_1^{-5/3}, \tag{17a}$$

$$\phi_{22}(k) \sim k_1^{-5/3}, \tag{17b}$$

$$\phi_{33}(k) \sim k_1^{-5/3}. \tag{17c}$$

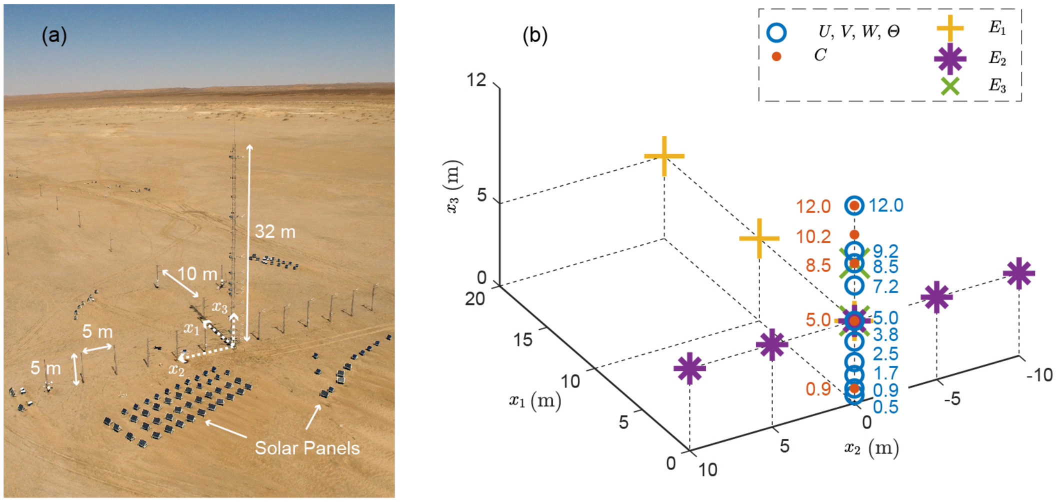

Supplementary Figure 1. **Overview of the measurement array.** (a) The coordinate defined in this study. The x_1-, x_2-, and x_3-axis are defined along the prevailing wind (i.e., streamwise) direction, spanwise direction, and vertical direction, respectively. The 5-m-height observation towers are evenly spaced 5 m apart in the spanwise direction (i.e., x_2), but they are spaced 10 m apart between the first three towers in the streamwise direction (i.e., x_1). Solar panels are used to charge the batteries that power all instruments. (b) The measurement array in this study. Here, the wind velocity and ambient temperature (i.e., U, V, W, and Θ) are denoted by circles (i.e., $\circ$), the PM10 dust concentration (i.e., C) are denoted by filled dots (i.e., $\bullet$), the streamwise components of the electric field (i.e., E_1) are denoted by crosses (+), the spanwise components of the electric field (i.e., E_2) are denoted by stars (i.e., $*$), and the vertical components of the electric field (i.e., E_3) are denoted by crosses (i.e., $\times$). The wind velocity and ambient temperature were measured at ten heights along the x_3-axis (i.e., $x_3 = 0.5$, 0.9, 1.7, 2.5, 3.8, 5.0, 7.2, 8.5, 9.2, and 12.0 m, with $x_1 = 0$ and $x_2 = 0$). The PM10 dust concentrations were measured at five heights (i.e., $x_3 = 0.9$, 5.0, 8.5, 10.2, and 12.0 m, with $x_1 = 0$ and $x_2 = 0$). The streamwise components of the electric field were measured at three locations along the x_1-axis (i.e., $x_1 = 0$, 10, and 20 m, with $x_2 = 0$ m and $x_3 = 5$ m). The spanwise components of the electric field were measured at five locations along the x_2-axis (i.e., $x_2 = -10$, -5, 0, 5, and 10 m, with $x_1 = 0$ m and $x_3 = 5$ m). The vertical component of the electric field was measured at two locations along the x_3-axis (i.e., $x_3 = 5$ and 8.5 m with $x_1 = 0$ and $x_2 = 0$ m).

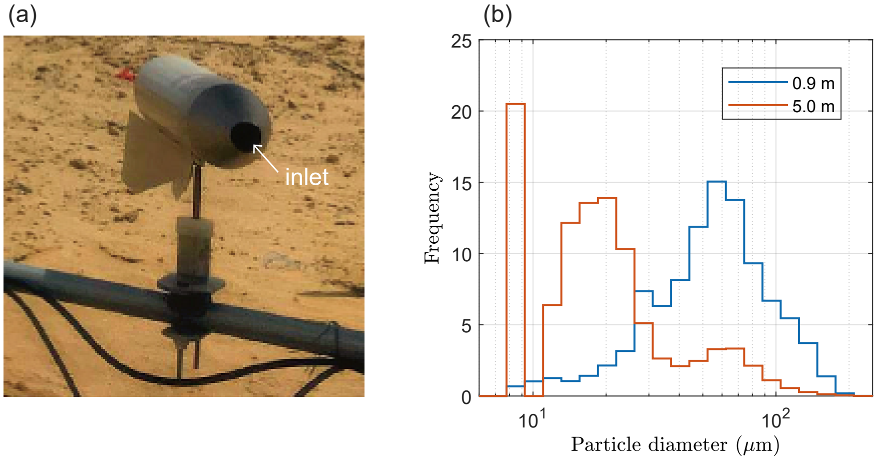

Supplementary Figure 2. **Number distributions of the dust particles collected during one week (i.e., from 15 April to 21 April, 2017, where the severe and mild dust storm datasets were extracted).** (a) A dust collector was mounted on 0.9 m above the surface. This photograph is adapted from Supplementary Fig. 7a in Ref.[8]. (b) Number distributions of the dust particles collected by the dust collectors at 0.9 m and 5 m heights were determined by a laser particle size analyzer (S3500, Microtrac Inc.). Let discrete random variable D be the particle diameter (in μm), and define the probability number function $p(d_i)$ of D by $p(d_i) = P\{D = d_i\}$. Thus, we can compute the particle volume fraction as $\frac{C_{10}}{\rho_p}\frac{\sum_i p(d_i)d_i^3}{\sum_{d_i \leqslant 10} p(d_i)d_i^3}$, where ρ_p and C_{10} denote the PM10 mass concentration and the mass density of the dust particles, respectively. Similarly, the particle-to-air mass loading ratio can be computed as $\frac{C_{10}}{\rho}\frac{\sum_i p(d_i)d_i^3}{\sum_{d_i \leqslant 10} p(d_i)d_i^3}$, where ρ denote the air density.

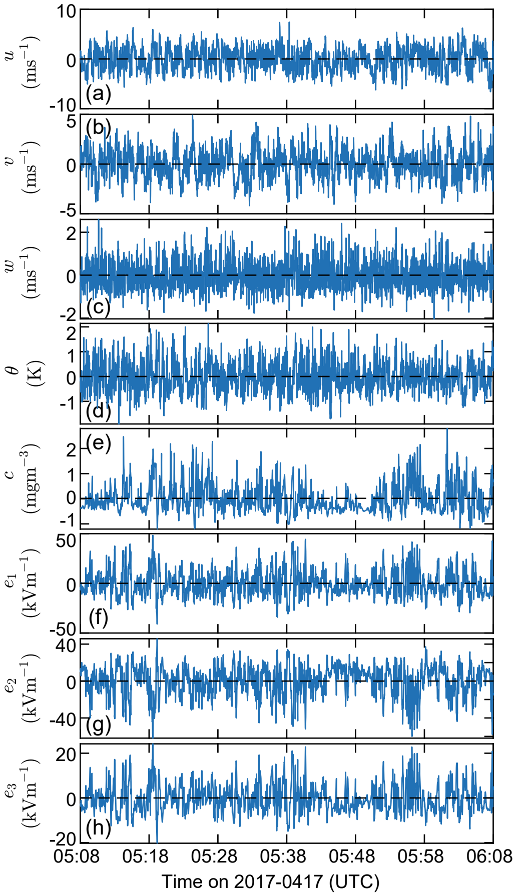

Supplementary Figure 3. **The filtered fluctuating fields for the observed severe dust storm dataset.** (a)-(h) Top-to-bottom panels, in turn, correspond to the filtered fluctuating fields of streamwise wind speed (i.e., u), spanwise wind speed (i.e., v), vertical wind speed (i.e., w), ambient temperature (i.e., θ), PM10 dust concentration (i.e., c), streamwise component of the electric field (i.e., e_1), spanwise component of the electric field (i.e., e_2), and vertical component of the electric field (i.e., e_3), respectively. For clarity, these data are plotted with a frequency of 1 Hz; that is, the wind velocity and ambient temperature data were resampled by averaging fifty data points of the original data to form one data point in the final data.

SUPPLEMENTARY REFERENCES

[1] Shao, Y. *Physics and Modelling of Wind Erosion* (Springer, Dordrecht, 2008).
[2] Maxey, M. R. *J. Fluid Mech.* **174,** 441-465 (1987).
[3] Stull, R. B. *An introduction to boundary layer meteorology* (Springer, Dordrecht, 1988).
[4] Warhaft Z. *Annu. Rev. Fluid Mech.* **32,** 203-240 (2000).
[5] Alexakis, A. & Biferale, L. *Phys. Rep.* **767-769,** 1-101 (2018).
[6] Landau, L. D. & Lifshitz E. M. *The classical theory of fields* (Elsevier, Oxford, 1975).
[7] Pope, S. B. *Turbulent Flows* (Cambridge Univ. Press, Cambridge, 2000).
[8] Zhang, H. & Zhou, Y. H. Reconstructing the electrical structure of dust storms from locally observed electric field data. *Nat. Commun.* **11,** 5072 (2020).
